# Recombinant PBP2a/autolysin conjugate as PLGA-based nanovaccine induced humoral responses with opsonophagocytosis activity, and protection versus methicillin-resistant *Staphylococcus aureus* infection

**DOI:** 10.22038/IJBMS.2022.59992.13303

**Published:** 2022-04

**Authors:** Setareh Haghighat, Seyed Davar Siadat, Abbas Akhavan Sepahi, Mehdi Mahdavi

**Affiliations:** 1 Department of Microbiology, Faculty of Advanced Science and Technology, Tehran Medical Sciences, Islamic Azad University, Tehran, Iran; 2 Department of Mycobacteriology & Pulmonary Research, Microbiology Research Center, Pasteur Institute of Iran, Tehran, Iran; 3 Department of Microbiology, Faculty of Basic Sciences, North Tehran Branch, Islamic Azad University, Tehran, Iran; 4 Advanced Therapy Medicinal Product (ATMP) Department, Breast Cancer Research Center, Motamed Cancer Institute, Academic Center for Education, Culture and Research(ACECR), Tehran, Iran; 5 Recombinant Vaccine Research Center, Tehran University of Medical Sciences, Tehran, Iran; 6 Immunotherapy Group, The Institute of Pharmaceutical Sciences (TIPS), Tehran University of Medical Sciences, Tehran, Iran

**Keywords:** Autolysin, Methicillin-resistant - Staphylococcus aureus, Nanovaccine, PBP2a, PLGA

## Abstract

**Objective(s):**

Methicillin-resistant *Staphylococcus aureus *(MRSA) reasons extreme infections, can resist various conventional antimicrobial agents, and cause morbidity and mortality worldwide. Vaccination seems to help modulate MRSA infections. Nanovaccine is considered a novel strategy in vaccine technology. The primary purpose of the present study was to develop a conjugate vaccine based on recombinant PBP2a and MRSA autolysin formulated in PLGA as a nanoparticle capable of enhancing protective responses against MRSA in the murine model.

**Materials and Methods:**

Recombinant PBP2a and autolysin have been expressed and purified by nickel-nitrilotriacetic acid (Ni-NTA) affinity column and characterized by SDS-PAGE and western blot. PLGA was bound to recombinant proteins by using 1-ethyl-3-(3-dimethylaminopropyl) carbodiimide hydrochloride (EDAC) and adipic acid dihydrazide (ADH) as a linker and spacer, respectively. Conjugation of recombinant proteins to PLGA was confirmed by the AFM assay, zeta potential, and size distribution, and its efficacy was evaluated in mice. Total IgG, IgG1, IgG2a, IgG2b, and IgM titers were analyzed to assess immune responses. Lastly, the bioactivity of antibodies was tested by using the opsonophagocytosis assay.

**Results:**

Mice immunized with the r-PBP2a-r-autolysin–PLGA nanovaccine led to increased levels of opsonic antibodies and IgG1, IgG2a, IgG2b, and IgM when compared with other experimental groups. Our results confirmed that vaccination with nanovaccine could reduce the mortality rate against the sub-lethal dose of MRSA challenge. Furthermore, the nanovaccine could eliminate MRSA from the kidney of infected mice.

**Conclusion:**

This study may provide valuable insights into the protective power of the r-PBP2a-r-autolysin–PLGA conjugate vaccine against MRSA infection.

## Introduction

Nanotechnology plays a pivotal role in vaccine design (1, 2). In parallel, nanovaccines are considered a distinctive approach to vaccination methodology (3). Nanoparticles are fabricated in microspheres, nanobeads, or micro-nano projections (4). As a hot topic in vaccine technology, nano vaccination has recently attracted enormous interest (5, 6). Several nanoparticle vaccines varying in chemical structure, dimension, morphology, and surface modifications have been explored for human applications, and the number of potential candidates is rising (2, 7).

Poly lactic-co-glycolic acid (PLGA) is one of the foremost applied biodegradable polymers because its hydrolysis results in metabolite monomers like lactic acid and glycolic acid(8, 9). Because those two monomers are endogenous and commonly metabolized by using the human body via the Krebs cycle, the systemic side effects are related to the utilization of PLGA for drug delivery or medicinal implementations (1). PLGA is accepted by the U.S. Food and Drug Administration (FDA) and European Medicine Agency (EMA) as a kind of drug delivery platform in humans (10-12). The polymers are commercially used with different molecular weights and chemical compositions(10, 13). PLGA-nanoparticles are infiltrated in cells marginally through fluid-phase pinocytosis and clathrin-mediated endocytosis. PLGA-nanoparticles escape the endo-lysosomes and enter the cytoplasm at intervals of 10 min of incubation (14). This reaction of nanoparticles with the vesicular membranes leads to the membrane’s transient colocalization followed by the escape of nanoparticles into the cytosol. The body distinguishes hydrophobic patches as foreign. The reticuloendothelial system (RES) removes hydrophilic particles from the blood and takes them up within the liver or the spleen(13). This route is one of the most pivotal biological mechanisms for nanoparticle-mediated controlled drug release. Attachment of opsonin proteins of plasma onto the nanoparticle surface results in binding opsonized materials to macrophages, consequently resulting in their internalization by phagocytosis (15, 16). Surface modification of nanoparticles can also demonstrate a significant impact on their interaction with cells and their internalization. PLGA nanoparticles show a negative charge distribution that may be changed with neutral or positive groups by surface modification, like PEGylation of the PLGA polymer (15, 17) or chitosan(18) coating, respectively.

Methicillin-resistant *Staphylococcus aureus* (MRSA) infections remain a significant health problem and are a number one reason for substantial morbidity and mortality (19, 20). There are various virulence factors concerned with infection and antibiotic resistance; autolysin (Atl) is one of the most vital virulence factors. Bacterial autolysins are potentially lethal enzymes that hydrolyze the peptidoglycan compounds of the cell wall and are included in the separation of daughter cells after cell division (21, 22). Furthermore, the mechanism by which *Staphylococcus aureus* develops antibiotic resistance comprises changes in penicillin-binding protein 2a (PBP2a) (23-25). Previous studies on recombinant proteins Atl and PBP2a showed their protective roles in mouse models (22, 26-29). Because adjuvants constitute decisive materials for vaccines, the discovery of more efficient adjuvants may permit the design and organization of prophylactic and therapeutic vaccines against infectious diseases like MRSA (28, 30). Adjuvants can increase vaccine efficacy through various known and unknown mechanisms as a helper factor (31).

Here, we hypothesized that r-PBP2a-r-autolysin conjugates formulated in a nanovaccine structure using PLGA could increase vaccine immunogenicity and efficacy. To this end, experimental mice were immunized with candidate vaccines, and then the humoral responses, opsonic killing, and protective responses were assessed in the mice.

## Materials and Methods

***Recombinant autolysin and PBP2a preparation***

As described previously, recombinant autolysin and PBP2a proteins were expressed and purified as histidine-tagged proteins in a bacterial expression system (22, 27). The recombinant proteins were subsequently purified under denaturing conditions using Ni-NTA affinity chromatography. The purified proteins were then analyzed using SDS-PAGE (12%) and western blot, dialyzed against phosphate-buffered saline (PBS), and finally quantified by the Bradford protein assay. 

***Preparation of r-autolysin-r-PBP2a conjugate***

According to the method described previously (26, 32), the conjugation of r-autolysin-r-PBP2a was carried out. In summary, each recombinant protein (1 mg) was dissolved separately in 2 ml of PBS-TIF (PBS-Trifluoroacetic acid) (0.5%, w/v) buffer. Then, solutions of anidric acetic acid in triethanolamine (5 mg/ml), 1-ethyl-3-(3-dimethylaminopropyl)-carbodiimide (EDAC) (5 mg/ml), and N-hydroxysuccinimide (NHS) (1 mg/ml) was added to the dissolved solutions and mixed well. The resulting solutions were stirred at 18–25 °C for 4 hr. After stirring, adipic acid dihydrazide (ADH) (0.5 M ﬁnal concentration) was added, and the reaction mixture was stirred at 18–25 °C for 6 hr. The r-autolysin solution was then added to the r-PBP2a solution and incubated at 25 °C for 3–4 hr. Afterward, the resulting mixture was subjected to dialysis against PBS buffer and after filtration and Bradford assay stored at -20 °C until used.

***Preparation of PLGA nanoparticles***

For fabrication of PLGA nanoparticles, a solvent diffusion method was performed. Briefly, first, dissolving of 50 mg PLGA in 3.0 ml acetone was implemented. Then, the obtained solution was added to 30 ml of 0.2% poly (ethylene-maleic acid) (PEMA) and stirred at 200 rpm, respectively. The PEMA surfactant enhanced carboxyl moieties on the surface of the particle. At the next stage, the collected fabricated nanoparticles were washed, and using a Zetasizer the size and zeta potential of the PLGA nanoparticles were investigated. 

***Synthesis of PEGylated-PLGA nanoparticles linked to conjugated proteins***

 Fmoc-PEG3400-COOH was stirred in 1.5 ml of 20% piperidine in DMF for 2 hr at ambient temperature for amino deprotection. Then, centrifugation and filtration (MWCO 5,000) were applied to the sample and caused the elimination of essentially complete Fmoc. The solution was dialyzed in 500 MWCO Float-A-Lyzers earlier than being lyophilized at the next stage. Then, incubation of 5 mg of PLGA nanoparticles in suspension (~0.2 mg/ml in double-distilled water) with 23 mg N-hydroxysuccinimide (NHS, 0.2 mmol) was applied at pH 5.8. 153 mg of 1-(3-dimethylaminopropyl)-3-ethylcarbodimide hydrochloride (EDAC, 0.8 mmol) was then added and incubated for 2 hr at ambient temperature with mild stirring. The connection between newly-synthesized NHS-activated particles and 10 mg NH_2_-PEG-COOH was carried out covalently bound. After fabrication of PLGA-PEG-COOH nanoparticles, washing and resuspending were implemented, and obtained nanoparticles were preserved in suspension form in double-distilled water. PLGA-PEG-COOH nanoparticles have been activated via NHS/ EDAC as defined above. The produced NHS-activated particles were covalently connected to 10 mg conjugated protein. Centrifugation at 10000 rpm for 30 min was carried out to separate the resulting PLGA-PEG-r-PBP2a-r-autolysin-COOH nanoparticles. The AFM assay confirmed the conversion of recombinant proteins to PLGA, zeta potential, and size distributions.

***Animals and immunization***

Six- to 8-week-old female BALB/c mice (20–25 g), classified into 9 experimental groups (each containing 14 mice), were used for the immunogenicity assay. All animal experiments were approved by the institutional animal care and ethics committee of Pasteur Institute of Iran. 

Mice were immunized three times at 21 day intervals as follows: 

Group1: r-PBP2a-r-Autolysin-PLGA conjugate (5 µg),

Group 2: r-PBP2a-r-Autolysin-PLGA conjugate (20 µg),

Group 3: r-PBP2a-r-Autolysin –Alum

Group 4: r-PBP2a-PLGA conjugate (20 µg),

Group 5: r- Autolysin-PLGA conjugate (20 µg),

Group 6: r-PBP2a-Alum,

Group 7: r- Autolysin-Alum

Group 8: r PLGA (20 µg; as a control group), and

 Group 9: PBS (as a control group).

Blood samples were collected from the retro-orbital sinus three weeks after each immunization, and the sera were stored at -20 °C until used.

***Evaluation of total IgG and specific IgG1/IgG2a/IgG2b and IgM isotypes***

An optimized indirect ELISA method evaluated specific total IgG antibodies, IgG isotypes (IgG1, IgG2a, and IgG2b), and IgM. Briefly, the 96-well ELISA plates (Greiner, Germany) were coated overnight with 100 μl of each recombinant protein or conjugates (1 μg/well) at 4 °C and subsequently blocked with 5% skimmed milk in PBS (as blocking buffer). Next, the sera diluted in blocking buffer (1:100 to 1:12,800) were added to the plates, followed by the 1:10,000 dilution of HRP-conjugated anti-mouse IgG, IgG1 IgG2a, IgG2b, and IgM (Sigma, USA) as secondary antibodies. After washing, the plates were incubated with the Tetramethylbenzidine (TMB) substrate, and antibody reactivity was determined at 450 nm using an ELISA reader (Awareness Stat Fax 2100, USA).

***Opsonophagocytosis assay***

The opsonophagocytic assay of experimental sera was carried out as described previously (22, 33). The test included *S. aureus* strain COL (OD = 0.2; at 650 nm) (~10^8^ CFU/ml in 1% BSA); mouse macrophages (2 × 10^7^/ml); diluted serum samples (1:2 to 1:16) and 4% baby rabbit serum. Evaluation of the opsonic killing activity of the immune sera was compared with those of the pre-immune serum. The test was conducted in triplicate for each quantity. The percent opsonic activity of the serum was calculated as follows:

Percentage of killed bacteria= [1- (CFU of Immune serum/CFU of Preimmune serum)] ×100

***Experimental challenge***

Three weeks after the last immunization, the immunized mice were infected by *S. aureus* COL 5×10^8^ CFU (sub-lethal dose) of bacteria (three times the LD_50_ dose optimized in our laboratory) through intraperitoneal injection. Afterward, the survival rate of the animals was regularly monitored for 30 days after the bacterial challenge.

***Bacterial inoculum in the kidney***

To assess bacterial burden in the kidneys, four mice were sacrificed 72 hr after systemic infection with *S. aureus* COL strain (approximately 5 × 10^8^CFU). In aseptic conditions, kidney samples were harvested and homogenized in sterile saline. Lastly, serial dilutions of homogenates were plated onto the L.B. agar, containing 50 µg/ml of oxacillin in duplicate, and CFU was enumerated after 24 hr of incubation at 37 °C. Results were calculated as log CFUs per gram of infected organs.

***Statistical analysis***

Data from immune responses were statistically analyzed using One Way Analysis of Variance (ANOVA) followed by Tukey HSD tests. Kaplan–Meier survival curves and the log-rank test were used for challenge experiments using version 8 Prism (GraphPad Software, San Diego, CA, USA) program. All data in this study were expressed as mean ± SD. *P*-values less than 0.05 were considered to be statistically significant.

## Results

***Protein expression and puriﬁcation***

**Figure 1:**
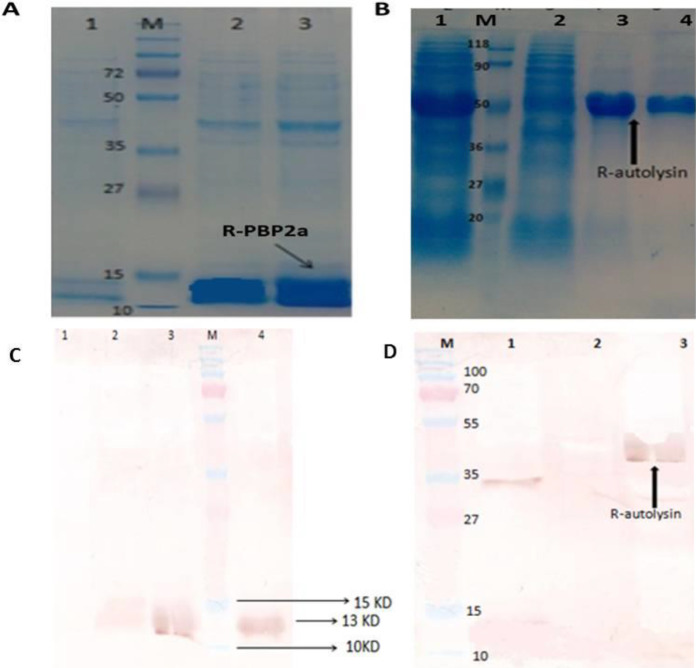
Purification of expressed r-PBP2a and r- autolysin proteins by the Ni-NTA column on SDS-PAGE (12% w/v) and western blot with anti-Atl anti-His (1:1000)

Results from SDS-PAGE analysis revealed that the highest amount of r-autolysin and r-PBP2a proteins were overexpressed in *E. coli* BL21 (DE3) through the induction of 1 mM IPTG at 37 °C for 6 hr. The molecular sizes of the expressed proteins were found to be approximately13 kDa and 43 kDa, respectively. The recombinant proteins were purified under a denaturation condition using Ni-NTA affinity chromatography (Figures 1A and 1B). Western blotting was used for protein analysis and veriﬁcation (Figures 1C and 1D). 

***Nanoparticle analysis***

**Figure 2:**
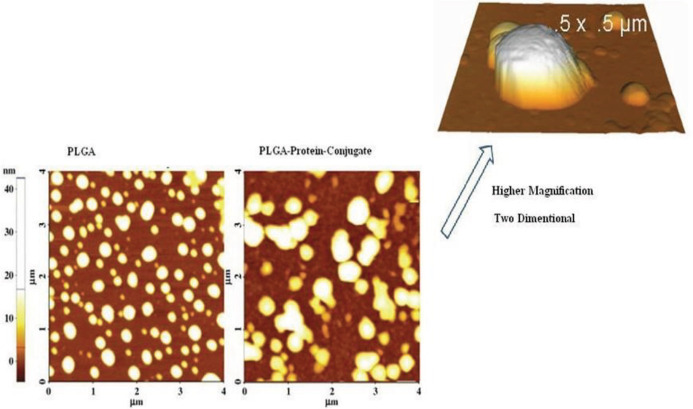
AFM images of the Poly lactic-co-glycolic acid (PLGA) and PLGA-PBP2a-autolysin conjugate

The size and zeta potential of nanoparticles was measured by the device Zeta Sizer (Malvern / U.K.). As shown in Table 1, different recombinant proteins (PBP2a, autolysin, and PBP2a-autolysin) were added to the PLGA nanoparticles, causing the enhanced dimension of the particles. Based on the zeta potential values, the addition of autolysin, PBP2a, and conjugate PBP2a-autolysin recombinant proteins made nanoparticles’ zeta potential more negative than PLGA, demonstrating conjugation of the above-mentioned recombinant proteins to the PLGA nanoparticles. Additionally, the AFM images of the PLGA and conjugate were taken and compared with each other, as shown in Figure 2.

***Total IgG antibodies***

An optimized indirect ELISA investigated specific total IgG. Results of assessing total specific IgG antibodies in the experimental groups indicated a significant increase of this factor in all vaccine-immunized groups (groups 1–7) compared with control ones (groups 8 and 9 ) (*P*<0.0001). 

**Figure 3:**
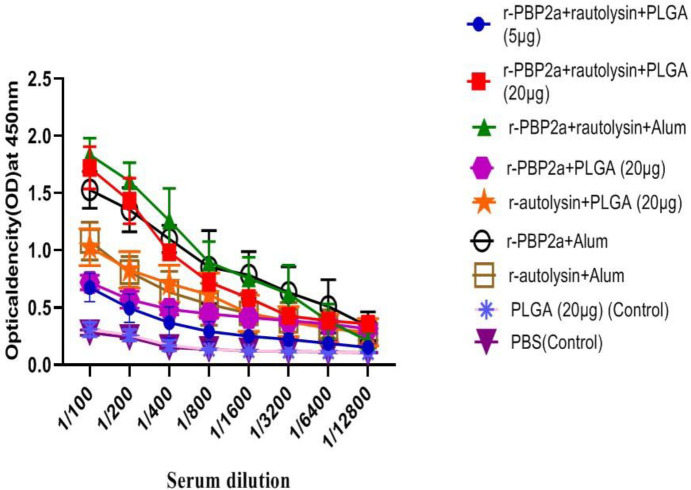
Titration of specific total IgG in experimental groups using indirect ELISA. Sera from different groups were diluted (1/100 to 1/12800), followed by an ELISA assay assessment. The results are the average of three independent experiments. Values are presented as mean ± SD of 14 mice in each group

Outcomes from titration in the experimental groups after the third immunization demonstrated that the total antibodies at a dilution of 1/100 to 1/1600 in mice immunized with r-autolysin-r-PBP2a-PLGA conjugate (20 μg), r-autolysin-r-PBP2a-Alum, and r-PBP2a-PLGA raised significantly, as compared with the other experimental groups (*P*<0.0001) (Figure 3). 

There was no significant difference in total IgG at any dilution between r-autolysin-r-PBP2a-PLGA conjugate mice (20 μg) and r-autolysin-r-PBP2a-Alum adjuvant groups (*P*>0.05).

The IgG response in mice that received r-autolysin with Alum adjuvant and r-autolysin-PLGA showed signiﬁcant differences versus mice that received r-autolysin-r-PBP2a-PLGA conjugate (5 μg) until dilution of 1/1600 (*P*=0.0187 and *P*=0.0090, respectively). However, total IgG in any dilution was not significantly different between r-autolysin-Alum and r-autolysin-PLGA (*P*>0.05). Also, there was a remarkable increase in the vaccine candidate group formulated with r-PBP2a - Alum as compared with r-autolysin-r-PBP2a-PLGA conjugate (5 μg) and r-PBP2a-PLGA vaccine groups until dilution of 1/3200 (*P*=0.0019 and *P*<0.0001, respectively).

Immunization with r-autolysin-PLGA induced specific antibody until dilution of 1/400 shows significant differences compared with r-PBP2a -PLGA (*P*=0.0038). Nevertheless, the speciﬁc total IgG levels of mice were vaccinated with r-PBP2a-Alum until dilution of 1/3200 was higher than r-autolysin-Alum (*P*=0.0019).

***Specific IgG1/IgG2a/IgG2b and IgM isotypes*** ***analysis***

The types of immune responses to recombinant proteins and conjugate were further assessed by evaluating the levels of three subtypes IgG (IgG1, IgG2a, and IgG2b) and IgM isotypes.

**Figure 4:**
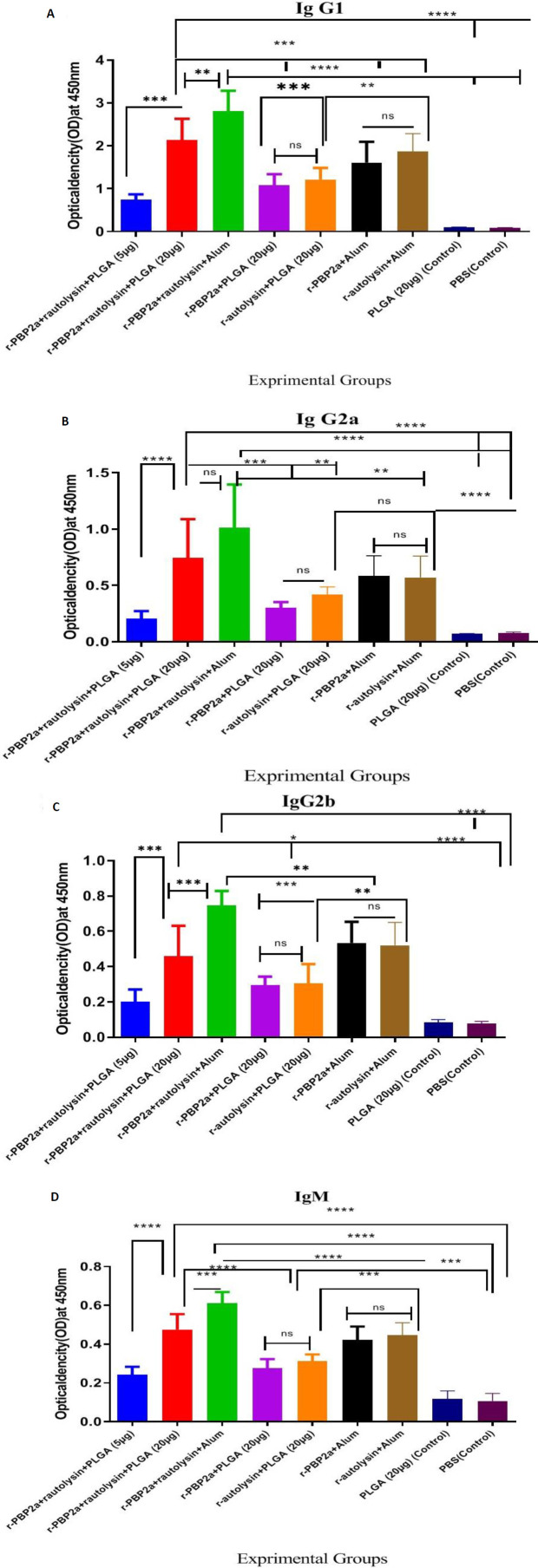
Levels of specific IgG1, IgG2a, IgG2b, and IgM in 28-day serum from vaccinated mice. Analysis of IgG1 (A), IgG2a (B), IgG2b(C), and IgM (D) titers shown in four distinct graphs. Each mouse serum was analyzed in duplicate by indirect ELISA using anti-IgG1, anti-IgG2a, anti-IgG2b, and anti-IgM antibodies and conjugated secondary antibody (HRP). Values for individual isotypes are expressed in OD_450_ nm (Mean ± SD)

As shown in Figure 4A, all experimental groups (groups 1–7) had the dramatically enhanced IgG1 isotope in comparison with the control groups (groups 8 and 9) (*P*<0.0001). Mice immunized with a 20 μg dose of the nanovaccine conjugate (group 2) had the markedly enhanced IgG1 isotype compared with group 1 receiving a 5 μg dose of the nanovaccine conjugate, groups 4 and 5 with 20 μg of the recombinant protein conjugate PLGA alone (*P*=0.0090, *P*=0.0004, and *P*=0.0057, respectively). 

The mice immunized with r-autolysin-PLGA and r-PBP2a-PLGA (*P*=0.9965) did not have any significant differences in IgG1 isotypes. Also, the IgG1 level did not have any significant difference in the group immunized with r-autolysin-PLGA and r-PBP2a-PLGA compared with group 1 receiving a 5 μg dose of the nanovaccine conjugate (*P*=0.1421 and *P*=0.5570, respectively).

Active immunization of r-autolysin-r-PBP2a with the Alum adjuvant showed a high level of IgG1 in comparison with r-PBP2a-Alum(group6) and r-autolysin-Alum(group7) groups (*P*<0.0001). However, there was no static difference between r-PBP2a-Alum and r-autolysin-Alum immunized groups (*P*=0.8342); also, remarkable differences were detected between r-autolysin-r-PBP2a -Alum and r-autolysin-r-PBP2a –PLGA (20μg) groups in the induction of specific IgG1isotype (*P*=0.0056).

The groups of mice that received r-autolysin formulated in Alum adjuvant had higher level of IgG1isotype versus r-autolysin-PLGA and r-PBP2a-PLGA groups (*P*=0.0081 and *P*=0.0006, respectively). However, there was no significant difference in the level of IgG1 between r-PBP2a-PLGA and r-PBP2a-Alum groups (*P*=0.0665).

Significant differences were observed in IgG2a isotype in all vaccinated groups (groups 1–7)

(Figure 4B) in comparison with the control groups (groups 8 and 9) (*P*<0.0001).

Mice immunized with a 20 μg dose of the nanovaccine conjugate (group 2) had the markedly enhanced IgG2a isotype in comparison with group 1 receiving a 5 μg dose of the nanovaccine conjugate, r-PBP2a-PLGA and r-autolysin-PLGA (groups 4 and 5) (*P*<0.0001, *P*=0.0009, and *P*=0.0347, respectively). There was not any statistical difference in the IgG2a level between the group immunized with r-autolysin-PLGA and r-PBP2a-PLGA compared with r-PBP2a-r-autolysin-PLGA (5 μg**) (***P*=0.4444 and *P*=0.9865, respectively). There was no significant difference in IgG2a level between r-autolysin-PLGA and r-PBP2a-PLGA groups (*P*=0.9580).

The IgG2a in the r-autolysin-r-PBP2a –Alum immunized group showed an increase as compared with the r-PBP2a-Alum and r-autolysin-Alum groups (*P*=0.0016 and *P*=0.0010, respectively), but there was no significant difference in IgG2a level between r-PBP2a-Alum and r-autolysin-Alum groups (*P*>0.9999); in addition, no significant differences were observed between the r-autolysin-r-PBP2a -Alum and r-autolysin-r-PBP2a –PLGA (20 μg) groups in induction of the specific IgG2a isotype (*P*=0.1691).

There was no significant difference in IgG2a level among r-autolysin-Alum and r-autolysin-PLGA groups (*P*=0.8181). Also, there was no statistical difference in IgG2a levels between the group receiving r-PBP2a-Alum and r-PBP2a-Alum (*P*=0.1132).

Assessment of IgG2b in experimental groups showed an increase in the order of IgG2a.

According to the obtained results, all vaccinated groups (groups 1–7) had enhanced IgG2b isotope as compared with the control groups (groups 8 and 9) (*P*<0.0001) (Figure 4C).

Immunization with a 20 μg dose of the nanovaccine conjugate (group 2) had enhanced IgG2b isotype in comparison with group 1 receiving a 5 μg dose of the nanovaccine conjugate r-PBP2a-PLGA and r-autolysin-PLGA (groups 4 and 5) (*P*<0.0001, *P*=0.0394, and *P*=0.0274, respectively). Also, a significant difference was observed between r-autolysin-r-PBP2a-PLGA(20 μg) and r-autolysin-r-PBP2a-Alum (*P*<0.0001). 

 No significant difference was observed in the IgG2b level of the group immunized with r-autolysin-PLGA and r-PBP2a-PLGA compared with the r-PBP2a-r-autolysin-PLGA group (5 μg**) (***P*=0.6184 and *P*=0.5063, respectively). IgG2b in the r-PBP2a r-autolysin-Alum immunized group increased compared with r-PBP2a-Alum and r-autolysin-Alum groups (*P*=0.0013 and *P*=0.0005, respectively). However, there was no significant difference in IgG2b level between r-PBP2a-Alum and r-autolysin-Alum groups (*P*>0.9999).

Significant differences were detected between r-autolysin-r-PBP2a -Alum and r-autolysin-r-PBP2a –PLGA (20μg) groups in the induction of specific IgG2a isotype (*P*=0.0056).

Sera from vaccinated mice with r-autolysin-Alum released a higher level of IgG2b compared with r-autolysin-PLGA and r-PBP2a-PLGA groups (*P*=0.0013 and *P*=0.0007, respectively). Also, there was a statistical difference in the level of IgG2b among r-PBP2a-PLGA and r-PBP2a-Alum groups (*P*=0.0003).

Furthermore, evaluation of IgM showed that all experimental groups had increased specific IgM compared with control groups (*P*<0.0001). The group of mice that received the r-PBP2a- r-autolysin-PLGA (20μg) induced a higher level of IgM versus r-PBP2a- r-autolysin-PLGA (5 μg), r-autolysin-PLGA, and r-PBP2a-PLGA groups **(***P*<0.0001).

The data illustrated no statistical difference in the IgM level of r-autolysin-PLGA and r-PBP2a-PLGA groups (*P*=0.9436). Also, there was no statistical difference in the IgM level between the group vaccinated with r-autolysin-PLGA and r-PBP2a-PLGA compared with the nanovaccine conjugate group (5 μg**) (***P*=0.9585 and *P*=0.2920, respectively).

IgM isotype in the r-autolysin-r-PBP2a –Alum immunized group showed a considerable increase compared with the r-PBP2a-Alum and r-autolysin-Alum groups (*P*<0.0001). Albeit there was no marked difference in IgM level among r-PBP2a-Alum and r-autolysin-Alum groups (*P*>0.9930); furthermore, a significant difference was observed between r-autolysin-r-PBP2a-PLGA (20 μg) and r-autolysin-r-PBP2a-Alum ( P=0.0002).

Mice immunized with both r-autolysin-Alum and r-PBP2a-Alum high induced levels of IgM (*P*<0.0001) in contrast with r-PBP2a-PLGA; in addition, statical differences were detected between r-autolysin-Alum and r-autolysin-PLGA groups in induction of specific IgM isotype (*P*=0.0003)(Figure 4D).

***Opsonophagocytic assay***

**Figure 5:**
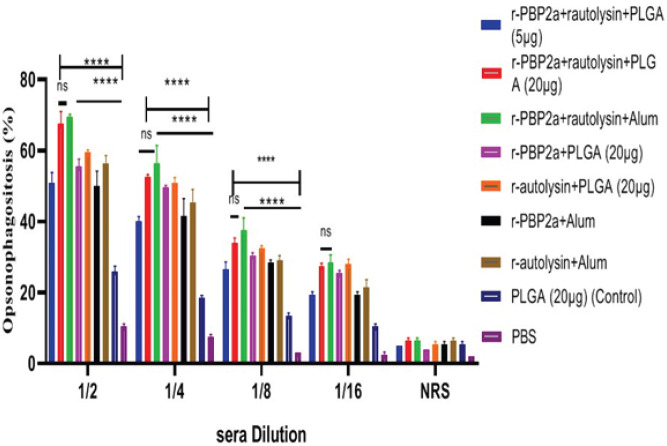
Comparative analysis of opsonic killing activity. Mouse macrophages dramatically killed the Staphylococcus aureus strain COL, which was opsonized with specific antibodies (at 1/4 to 1/16 dilution) raised against the recombinant proteins and conjugate, compared with the control groups. Bars represent the means of triplicate measurements, and the error bar indicates SD

The bioactivity of antibodies against recombinant proteins and conjugates to promote phagocytosis of bacteria were measured by using the incubation of *S. aureus* with pooled and diluted antibodies (pre-challenge serum) and mouse macrophages in the presence of rabbit complement. At dilutions (1/2 up to 1/16), the opsonic activity of vaccinated groups was remarkably higher in comparison with control groups (*P*<0.0001). Immunization with r-autolysin-r-PBP2a-PLGA (20μg) and r-autolysin-r-PBP2a-Alum remarkably increased phagocyte killing activity compared with other vaccinated groups (dilutions of 1/2 to 1/8, *P*<0.0001). There was no statistical difference in opsonic activity at any dilution (1/2 to 1/16) among r-autolysin-r-PBP2a-PLGA (20 μg) and r-autolysin-r-PBP2a-Alum (*P*>0.05). Serum dilutions (1:2 up to 1:16) of the mice immunized with r-autolysin-r-PBP2a-PLGA (20μg) and r-autolysin-r-PBP2a-Alum resulted in 67.5%–27.5% and 69.5%–28.5% opsonic killing activity, respectively (Figure 5).

***Quantiﬁcation of bacteria in the kidney after challenge***

**Figure 6:**
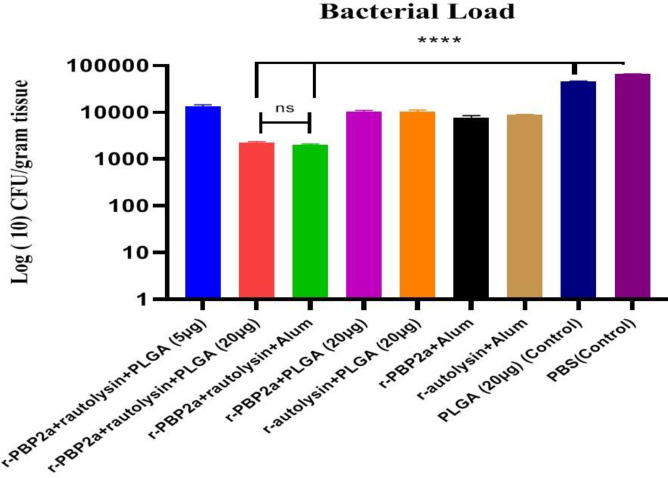
Functional activity of the specific immune antibodies induced against the recombinant proteins and conjugate. Two weeks after the last immunization, the mice were challenged with Staphylococcus aureus COL strain (5×10^8^ CFU) by intraperitoneal injection. Three days after the challenge, the bacterial burden was determined in the kidneys of the mice. The specific antibodies significantly decreased the bacterial loads in the infected tissues compared with control groups. Bars represent the means of triplicate measurements, and the error bar indicates SD. * indicates a significant difference (*P*<0.05)

After intraperitoneal infection, the bacterial loads were investigated in the kidneys five days after the challenge to explore the impact of nanovaccine candidates on bacterial elimination from the kidneys of infected mice. The data displayed in Figure 6 exhibited that the bacterial loads in all vaccinated groups were considerably reduced versus the control groups (*P*<0.0001). Impressively, it was found that mice immunized with r-PBP2a-r-autolysin conjugate nanovaccine (20 µg) and r- PBP2a-r-autolysin-Alum strongly reduced bacterial loads in their kidneys, as compared with the other vaccinated groups (*P*<0.05). In addition, no statistical difference was observed among bacterial loads between mice in group 2 (r-autolysin-r-PBP2a –PLGA conjugate (20 µg) and group 3 (r- PBP2a-r-autolysin-Alum) (*P*>0.9999). Overall, these data suggested that conjugation of the r-PBP2a-r-autolysin to PLGA as a nanovaccine candidate was more potent in the elimination of *S. aureus* from kidneys of infected mice as well as r- PBP2a-r-autolysin-Alum, when compared with other experimental groups.

***Survival rate***

**Figure 7:**
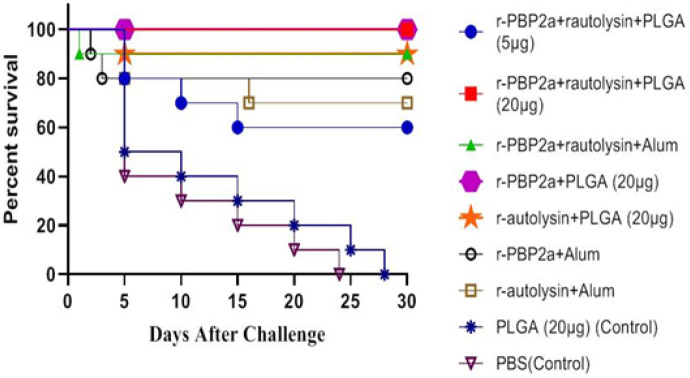
Protection of mice against sepsis infection by Methicillin-resistant *Staphylococcus aureus (*MRSA) (5 ×10^8^ CFU). Two weeks after the last immunization, the mice were infected intraperitoneally with Staphylococcus aureus COL strain. Their survival rate was recorded daily for 30 days. Mice immunized with r-autolysin-r-PBP2a-PLGA conjugate (20 µg), and r-PBP2a-PLGA (groups 2 and 3) demonstrated no mortality after challenge with the COL strain (100% survival)

Our results showed that the highest survival rates (100%) belonged to mice immunized with r-autolysin-r-PBP2a-PLGA conjugate (20 µg) and r-PBP2a-PLGA (groups 2 and 4), as compared with control groups (*P*<0.0001). The survival rate in mice immunized with r-autolysin-r-PBP2a-Alum and r-autolysin-PLGA was 90%. However, 20%, 30%, and 40% of the mice immunized with the r-PBP2a-Alum, r-autolysin-Alum, and r-autolysin-r-PBP2a-PLGA conjugates (5 µg), respectively were dead (Figure 7). 

A: Lane 1, the pellet of un-induced bacteria; lane2 and 3, purified r-PBP2a; lane M, standard protein size marker(kDa). B: lane 1, the pellet of IPTG induced bacteria, Lane 2, the pellet of un-induced bacteria; lane3 and 4, purified r-autolysin; lane M, standard protein size marker(kDa). C: Lane 1, Control negative (pET24a without mecA fragment) lane 2, the pellet of un-induced bacteria; lane 3 and 4 puriﬁed r-PBP2a; lane M, pre-stained protein size marker (kDa). D: Lane 1, the negative control (pET24a without the Atl fragment); Lane 2, pellet from non-induced bacteria; lane 3, puriﬁed r-autolysin; lane M, a pre-stained protein size marker (kDa). The r-PBP2a and r-autolysin proteins are shown by the arrow

**Table 1:** Typical size and Zeta potential distributions of the PLGA nanoparticles after the conjugation process. Concentration 0.5 mg/ml DDW and the experiments were repeated three times. The zeta potential data were presented as Mean ± SEM

Types	Z-Average (r.nm)	PDI	Zeta potential (mv)
PLGA-PEG	108.5nm	0.604	-39.07 ± 6.2
PBP2a-PLGA-PEG	185.4nm	0.612	- 42.12 ± 7.5
PBP2a-autolysin-PLGA-PEG	235.0 nm	0.622	-48. 33 ± 5.4

## Discussion

Subunits of pathogen-mediated modern vaccines, such as purified proteins, are not considered potential systems to evoke strong immune responses and need to be optimized for immunogenicity(34). One of the most crucial challenges in this area is the nomination of potential adjuvants and delivery systems for enhanced immunogenicity of purified proteins as vaccine candidates (35, 36). The best adjuvant candidate has to increase the antigen-specific immune responses and facilitate protection through stimulation of optimal kinds of immunity, in parallel to a rationale safety (37, 38). Studies showed that applying polymeric nanoparticles as adjuvants and delivery systems has many advantages, including their safety in human use and strong potency in inducing cellular and humoral immune responses (1, 39).

MRSA, an important pathogen that can cause serious infections, is usually resistant to conventional antimicrobial agents (40). Currently, vaccine efficacy against *S. aureus* and achievement of an efficient vaccine is of great importance resulting in protection in communities (40-42).

Herein, we hypothesized that formulation of the r-PBP2a-r-autolysin conjugate vaccine in the backbone of a nanostructure might increase vaccine immunogenicity, thereby leading to protective effects. In this regard, r-PBP2a-r-autolysin, as a recombinant protein, was formulated as a nanovaccine in the PLGA backbone and used as a novel MRSA vaccine candidate.

The impact of particle dimension on immunogenicity seems to be a consequence of increased uptake into the corresponding antigen-presenting cells (APCs) for the smaller-sized particles. The size range of nanoparticles used in nanovaccinology is between 2–1000 nm (1, 43), which was also confirmed in the current study. The size alteration of nanoparticles can confirm the addition of different recombinant proteins to the PLGA nanoparticle; our findings showed the appropriate size and zeta potential of the nanovaccine. As observed by AFM (Figure 2), the particle size of the conjugate was 235 nm which is suitable as a nanovaccine; in addition, the zeta potential of r-PBP2a-autolysin-PLGA-PEG was -48. 33 ± 5.4mv. It seems that the physical structure of the PLGA-based nanovaccine is a proper reconstitution for immunological purposes. 

In previous studies, the function of PLGA, as an adjuvant, in the increased secretion of interleukin-1β (IL-1β) via dendritic cells (D.C.s) has been reported (44-46). This mechanism can involve the increase of vaccines’ immunogenicity in this structure.

Colonna *et al*. have designed a unique adjuvanted system for vaccination against *S. aureus*-mediated infections. Mainly, poly-lactide-co-glycolide (PLGA) nanoparticles were developed to efficiently load and improve a sub-unit vaccine, specifically a purified recombinant collagen-binding bacterial adhesin fragment (CNA19). They demonstrated that mice immunized with CNA19 loaded PLGA induced a remarkable level of specific IgG antibodies compared with the control groups (47, 48).

Results of antibody response showed that mice immunized with nanovaccines induced higher antibody titer levels than the control groups. The highest levels of antibody titers were observed in group 2 (r-autolysin-r-PBP2a-PLGAconjugate) (20 μg) and group 3(r-autolysin-r-PBP2a-Alum). Previous reports in this field supported our results for the efficacy of PLGA in the stimulation of humoral immune responses (34, 39, 48, 49).

Analysis of antibody isotypes showed that the r-autolysin-r-PBP2a-PLGA conjugate induced the highest IgG1, IgG2a, IgG2b, and IgM antibodies at the dose of 20 µg, as compared with the other vaccinated groups. These results can somehow illustrate that PLGA is a potent adjuvant to induce poly-isotypic humoral immune responses, potentially neutralizing and eliminating pathogens. Attachment of antibodies with surface antigens of bacteria promotes neutralization and opsonization of pathogens. Regarding these mechanisms, each isotype of antibody may have a distinct function in the immune responses, and induction of poly-isotypic humoral immunity can show more potency of the humoral immune response. Notably, a remarkable increase was detected in both IgG2a and IgG2b isotypes after vaccination in r-autolysin-r-PBP2a-PLGA conjugate vaccine versus mere nano-vaccines and control groups which showed a Th1 pattern response. A variety of studies demonstrated that PLGA in the vaccine formulation could trigger cell-mediated immune responses(45, 50-52), which is essential in the clearance of the pathogen and induction of protectivity in parallel to the humoral immune response. Next, the bioactivity of raised antibodies showed a high phagocytosis activity (at 1:2 serum dilution) in the r-PBP2a-r-autolysin-PLGA conjugate (20 μg) and r-PBP2a-r-autolysin-Alum-immunized groups (67.5% and 69.5%) with a significant increase versus other vaccinated and control groups. An increase in humoral immune response and opsonic killing activity in the r-PBP2a-r-autolysin-PLGA conjugate (20 μg) vaccine group versus other experimental groups may result in a higher protectivity effect in the challenge study. 

In practice, survival rate results showed 100% protection in the r-PBP2a-r-autolysin-PLGA conjugate (20 μg) and r-PBP2a –PLGA groups compared with the other experimental and control groups. Despite the highest antibody response and opsonic killing activity of the r-PBP2a-r-autolysin-PLGA conjugate (20 μg) group, the survival rate of this group was the same as the r-PBP2a –PLGA group, which showed lower antibody and opsonic killing responses. This controversy may be due to the induction of other immunologic aspects that correlated with protection and were not assessed here.

Previous studies confirmed the efficiency of PBP2a within induction of humoral immune responses and safety against experimental MRSA infections (25, 27, 28, 53). Kalali *et al*. described that passive immunization with anti-r-autolysin IgG considerably reduced bacterial load inside the internal organs of mice after intraperitoneal challenge with a lethal dose of *S. aureus* COL strain (21).

In some other reports, the encapsulation of type III secretion system protein, PopB, and its chaperon molecule PcrH in PLGA nanoparticles might enhance Th17 responses to intranasal vaccination and protect mice against acute lethal *P. aeruginosa *pneumonia (54).

In our study, bacterial loads in the kidney of the vaccinated groups significantly decreased after intraperitoneal challenge compared with control groups.

The lowest bacterial load was observed in the r-PBP2a-r-autolysin-PLGA conjugate (20 μg) group, which suggested that conjugation of the r-PBP2a-r-autolysin to PLGA in the form of nanovaccine was more potent in elimination of *S. aureus* from the kidneys of infected mice versus other vaccinated groups. It seems that the r-PBP2a-r-autolysin-PLGA conjugate (20 μg) may induce better opsonic activity of challenged bacteria through dual antigen-targeting on the surface of MRSA and induction of more widespread isotypes of humoral immune responses versus r-PBP2a- PLGA and r-autolysin-PLGA groups, thereby provide better elimination of organisms with phagocyting cells. These advantages do not improve the survival rate of the r-PBP2a-r-autolysin-PLGA conjugate group versus r-PBP2a- PLGA and partly r-autolysin-PLGA groups, which may show the role of other immunologic factors.

## Conclusion

Our strategy to conjugate r-autolysin and r-PBP2a with PLGA nanoparticles as a nano-vaccine formulation can be introduced as a suitable vaccine candidate against MRSA infection in the mouse model. The vaccine-induced high levels of anti-autolysin and PBP2a antibody titers, a high percentage of opsonophagocytosis, improved the overall survival rate of vaccinated mice, and reduced *S. aureus *loads in the kidneys of the kidneys infected mice. Although our study showed promising findings through the nano-vaccination strategy, it is still preliminary, and more studies are needed to judge its usefulness and feasibility as a protective vaccine by finding other immunologic mechanisms involved in the protection.

## Authors’ Contributions

MM and SDS Conceived and designed the study; SH Performed data processing and collection, experiments, analysis and interpretation of results, draft manuscript preparation, and visualization; MM Critically revised and edited the article; SH, SDS, MM, and AAS Approved the final version to be published; SDS, MM, and AAS Supervised and helped with funding acquisition. 

## Conflicts of Interest

The authors declare no conﬂicts of interest associated with the present manuscript.
